# Application of Chatbots to Help Patients Self-Manage Diabetes: Systematic Review and Meta-Analysis

**DOI:** 10.2196/60380

**Published:** 2024-12-03

**Authors:** Yibo Wu, Jinzi Zhang, Pu Ge, Tingyu Duan, Junyu Zhou, Yiwei Wu, Yuening Zhang, Siyu Liu, Xinyi Liu, Erya Wan, Xinying Sun

**Affiliations:** 1 School of Public Health Peking University Beijing China; 2 School of Humanities and Social Sciences Harbin Medical University Harbin China; 3 Graduate School Harbin Medical University Harbin China; 4 School of Traditional Chinese Medicine, Beijing University of Chinese Medicine Beijing China; 5 School of Journalism and Communication, Hebei Institute of Communications, Shijiazhuang China; 6 School of Humanities and Social Sciences, University of Science and Technology of China Hefei China; 7 Institute of Communication Studies, Communication University of China Beijing China; 8 College of Nursing, North Sichuan Medical college Nanchong China; 9 College of Stomatology, Shandong University Jinan China; 10 School of Nursing and Rehabilitation, Xi’an Medical University Xi’an China; 11 School of Global Health, Shanghai Jiao Tong University Shanghai China

**Keywords:** artificial intelligence, chatbot, diabetes, health education, self-management, systematic review

## Abstract

**Background:**

The number of people with diabetes is on the rise globally. Self-management and health education of patients are the keys to control diabetes. With the development of digital therapies and artificial intelligence, chatbots have the potential to provide health-related information and improve accessibility and effectiveness in the field of patient self-management.

**Objective:**

This study systematically reviews the current research status and effectiveness of chatbots in the field of diabetes self-management to support the development of diabetes chatbots.

**Methods:**

A systematic review and meta-analysis of chatbots that can help patients with diabetes with self-management was conducted. PubMed and Web of Science databases were searched using keywords around diabetes, chatbots, conversational agents, virtual assistants, and more. The search period was from the date of creation of the databases to January 1, 2023. Research articles in English that fit the study topic were selected, and articles that did not fit the study topic or were not available in full text were excluded.

**Results:**

In total, 25 studies were included in the review. In terms of study type, all articles could be classified as systematic design studies (n=8, 32%), pilot studies (n=8, 32%), and intervention studies (n=9, 36%). Many articles adopted a nonrandomized controlled trial design in intervention studies (n=6, 24%), and there was only 1 (4%) randomized controlled trial. In terms of research strategy, all articles can be divided into quantitative studies (n=10, 40%), mixed studies (n=6, 24%), and qualitative studies (n=1, 4%). The evaluation criteria for chatbot effectiveness can be divided into technical performance evaluation, user experience evaluation, and user health evaluation. Most chatbots (n=17, 68%) provided education and management focused on patient diet, exercise, glucose monitoring, medications, and complications, and only a few studies (n=2, 8%) provided education on mental health. The meta-analysis found that the chatbot intervention was effective in lowering blood glucose (mean difference 0.30, 95% CI 0.04-0.55; *P*=.02) and had no significant effect in reducing weight (mean difference 1.41, 95% CI –2.29 to 5.11; *P*=.46) compared with the baseline.

**Conclusions:**

Chatbots have potential for the development of self-management for people with diabetes. However, the evidence level of current research is low, and higher level research (such as randomized controlled trials) is needed to strengthen the evidence base. More use of mixed research in the research strategy is needed to fully use the strengths of both quantitative and qualitative research. Appropriate and innovative theoretical frameworks should be used in the research to provide theoretical support for the study. In addition, researchers should focus on the personalized and user-friendly interactive features of chatbots, as well as improvements in study design.

## Introduction

### Background

Diabetes, as a significant global public health problem, has resulted in a heavy burden on the health care system. Approximately 530 million people worldwide currently have diabetes, and by 2050, more than 1.31 billion people will have diabetes globally [1]. In 2021, the global health expenditure caused by diabetes was about US $966 billion, and it is estimated that this figure will exceed US $1054 billion by 2045 [2]. Currently, there is no effective cure for diabetes, but it is somewhat controllable and preventable. The American Diabetes Association Professional Practice Committee suggests that diabetes may be diagnosed based on hemoglobin A_1C_ (HbA_1c_) criteria or plasma glucose criteria, either the fasting plasma glucose value, 2-hour glucose value during a 75-g oral glucose tolerance test, or random glucose value accompanied by classic hyperglycemic symptoms or hyperglycemic crises. Therefore, the main control of diabetes mellitus is the blood glucose level [3]. Diabetes control can reduce and delay the onset and progression of diabetes complications. Good diabetes control depends on a combination of diet, exercise, medication, diabetes education, and blood glucose monitoring [4]. However, high-quality treatment of diabetes and its complications also depends on education about diabetes and active management of the patient’s own behavior (also known as self-management) [5]. Health education is vital for the self-management of diabetes [6]. The purpose of diabetes education is to help patients improve their self-care ability, that is, to increase knowledge about self-care of diabetes and to improve the ability of self-care practice [5]. Diabetes self-management and support have been provided face-to-face (one-to-one or group based) for decades, with many trials and real-world studies showing improved diabetes outcomes [7]. However, high costs and resource requirements have limited the scope and scalability of face-to-face programs [8].

Digital therapeutics is a new concept: it is technology produced by the digital integration of medicine and health. It combines disease, data, and algorithms [9]. The essence of digital therapeutics is a disease intervention program based on evidence-based medicine and presented by software programs that provide core functions such as the prevention, treatment, or management of diseases and high-risk factors [5]. Digital therapy applications (Blue Star, Diabeo System, etc) use web-based applications or cloud platforms to provide evidence-based, personalized, and rapid care management for chronic and behavioral-changeable diseases, including diabetes [10]. Digital therapy applications can improve patients’ compliance, treatment success rate, and economic achievements in diabetes management by enhancing patients’ participation, changing patients’ lifestyles, providing comprehensive medical care, and regular glucose monitoring [11]. At present, the increase of patients with diabetes has brought a heavy burden to medical professionals and the medical care system. With the development of artificial intelligence (AI) and digital therapies, a convenient and effective diabetes health education system is gradually being developed [12]. It meets the individual needs of patients with diabetes to acquire knowledge about diabetes and self-management, helps patients improve their daily lifestyles, and supports them in the self-management of diabetes.

The chatbot is one of the products of scientific and technological progress and digital therapy, which has great potential in providing health education for patients and helping patients manage themselves. The chatbot can also be called a conversational agent or system, defined as a nonhuman virtual conversational bot, a computer program, or an AI program [13]. It participates in the conversation via audio or text input to answer the user’s questions, and the user can easily access the desired information by sending messages to the chatbot [14]. AI is now gradually being integrated into chatbots. When a user asks a question or issues a command in human language, the chatbot understands the contextual information sent by the user and responds accordingly. Google Assistant, Apple’s Siri, and ChatGPT are common chatbots with voice-activated interfaces [15,16]. Chatbots can play an essential role in many areas, especially in health care. They can help patients anytime via a smartphone app or the web [17]. Chatbots can provide fascinating health-related information and services and provide a personalized lifestyle [18]. Chatbots can usually provide better answers to health care questions than other health care resources [19]. For health care organizations, the use of chatbots significantly improves efficiency and reduces the workload of health care professionals and the burden on the health care system [20,21]. For patients, the use of chatbots can assist in self-management, improving behavioral compliance and quality of life [22]. Previous findings indicate that chatbot interventions effectively improve depression, anxiety, stress, medication adherence, and smoking cessation [23].

Chatbots have the potential to provide personalized health education and assist in self-management for people with diabetes. According to our findings, although some studies have analyzed diabetes and self-management or investigated the use of chatbots in other diseases [24,25], no study has systematically reviewed the use of chatbots in self-management for patients with diabetes. Therefore, this study is innovative to some extent.

### Objectives

In summary, the aim of this study was to conduct a systematic review and meta-analysis of chatbots used in the field of diabetes self-management, exploring the specific characteristics of chatbots, research methods, research strategies, evaluation measures of outcome indicators, and some other information. By analyzing information about chatbots, this study aims to assess the potential and effectiveness of chatbots in diabetes self-management, thus helping researchers to sort out the current state of research and future development trends in this field. This will provide support for improving the development of chatbots in the field of diabetes self-management, which will ultimately help patients with diabetes to stay healthy.

## Methods

### Search Strategy

A systematic search of the literature was conducted in the PubMed and Web of Science databases, from the date of database establishment until January 1, 2023. PubMed and Web of Science are the 2 most commonly used databases for scholars in the biomedical field. PubMed is the largest biomedical retrieval platform in the world. Web of Science is the most classic and authoritative citation database in the scientific community, and the most important core collection of Web of Science contains more than 12,000 academic journals in more than 250 disciplines. The quality of articles in these 2 databases is very high, and hence, the researcher chose to extract high-quality literature from these 2 databases. Search terms were developed from a combination of keywords and Boolean operators in consultation with a professional medical librarian. The keywords of the search mainly revolve around “diabetes” and “Chatbot” (see Table S1 in Multimedia Appendix 1 for the search strings).

### Data Management and Extraction

The data filtering process can be divided into four stages: (1) duplicate data removal, (2) primary filtering (filtering based on titles and abstracts), (3) secondary filtering (filtering based on full text), and (4) cross-checking. Two researchers (JZZ and GP) independently screened the articles according to the inclusion and exclusion criteria (see Table S2 in Multimedia Appendix 1). In the first step, for articles that were both in the Web of Science and PubMed databases, only one of them was retained, and duplicates were removed. In the second step, irrelevant articles were initially filtered out by reading titles and abstracts. In the third step, articles that did not fit the topic of the study were excluded by carefully reading the full text for judgment. Finally, independent screening results from JZZ and GP were cross-checked to ensure the accuracy of article screening. For articles with screening disagreements, the disagreements were resolved through discussion with the senior author, YBW. The PRISMA (Preferred Reporting Items for Systematic Reviews and Meta-Analyses) 2020 flow diagram was used to visualize the comprehensive search process and the results obtained.

The quality of the included literature was evaluated by 2 researchers independently using the evaluation criteria for observational studies developed by the Agency for Healthcare Research and Quality [26]. The tool has a total of 11 entries, each of which contains 3 options: “Yes,” “No,” and “Unclear.” A score of 1 was given for “Yes” and 0 for “No” or “Unclear”; the total score was 11, with 0-3 as low quality, 4-7 as medium quality, and 8-11 as high quality. Disagreements were resolved through discussion between authors. For details of the quality evaluation results, refer to Table S3 in Multimedia Appendix 1.

### Outcomes of Interest

The basic information and data from the articles were extracted and checked according to a predesigned data extraction table. The data information extracted for each study includes (1) basic information about the article (author, country, and year of publication); (2) specific features of the chatbot (name, consultable content, AI technology, question and answer database data sources, and input and output methods); and (3) research design aspects (research strategy, research type, theoretical framework, evaluation indexes of chatbot effect, etc).

### Meta-Analysis Methods

Meta-analysis was planned if the included studies were sufficiently homogeneous in terms of statistical characteristics. RevMan 5.3 software (Cochrane Collaboration) was used for the statistical analysis of data. Mean, SD, and 95% CI were used as study effect indicators. The heterogeneity test was performed on the combined data. If *P*>.10 and *I*^2^<50%, it suggests that the heterogeneity among studies was small, and the fixed-effect model was selected for meta-analysis. If *P*<.10 and *I*^2^>50%, it suggests that there is obvious heterogeneity among the studies, and the random effect model was used for analysis. Sensitivity analysis was performed by comparing the results of the combined effect sizes of the fixed-effects model and random-effects model to ensure the stability and reliability of the results [27].

## Results

### Search Results

**Figure 1:**
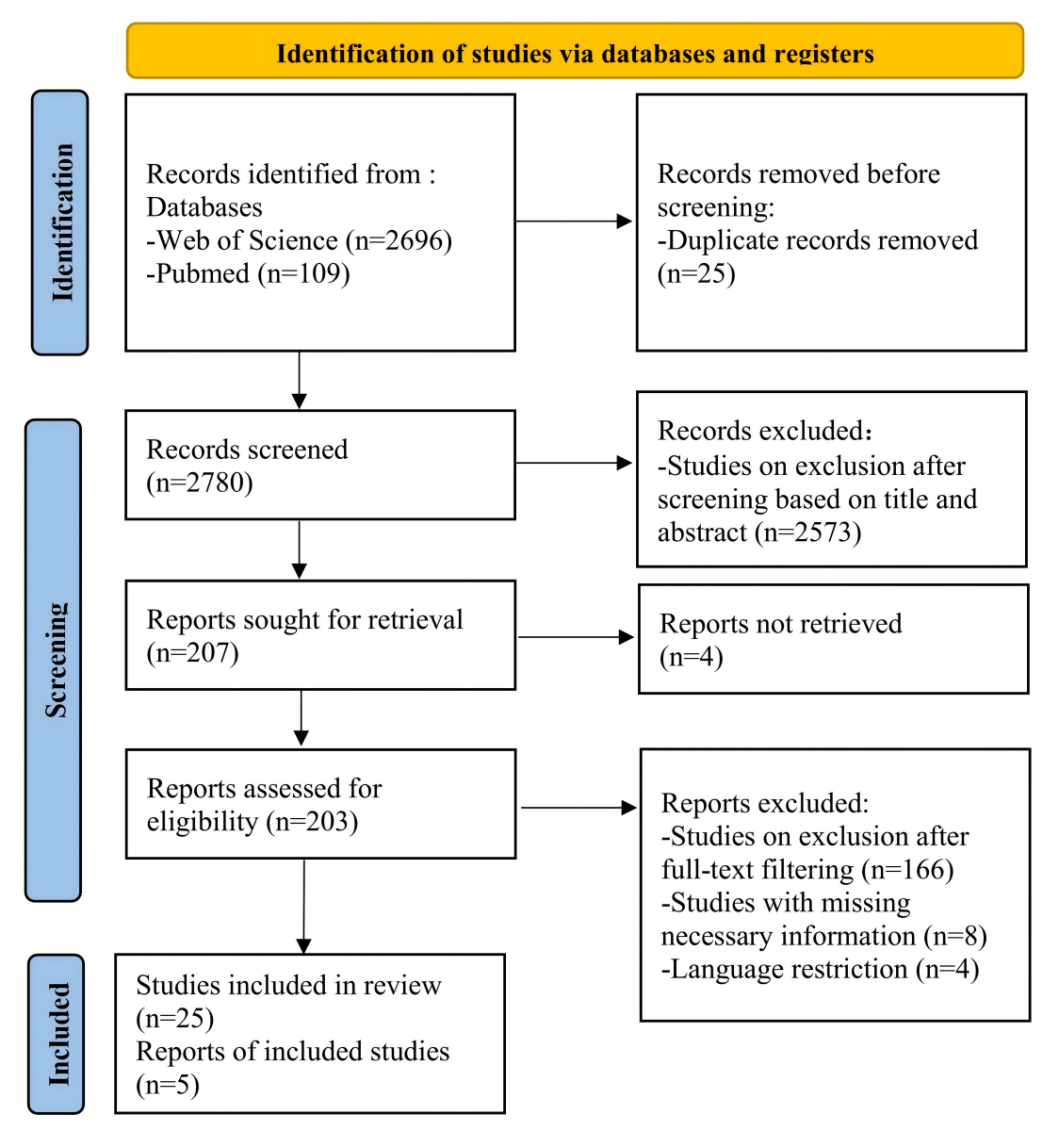
PRISMA (Preferred Reporting Items for Systematic Reviews and Meta-Analyses) flow diagram showing the study selection process.

In total, 25 articles from 14 countries were included in the analysis (see Table 1 for details). Authors from Australia and Portugal published 4 articles each, and authors from the United States and India published 3 articles each. Also, high-income countries, including Singapore, Norway, the Netherlands, Spain, Greece, and South Korea, and low-income countries, including China, Bangladesh, South Africa, and Ghana, have published relevant studies. The PRISMA flow diagram is shown in Figure 1.

**Table 1:** Basic information of included articles.

Study	Year	Research type	Country	Consultable content	Evaluation metrics
Krishnakumar et al [28]	2021	Interventional study	India	Diet, exercise, medications, and blood glucose	User experience and user health
Stephens et al [29]	2019	Pilot study	United States	Diet, exercise, and mental health	Technical performance and user experience
Sharma et al [30]	2018	System design	India	General diabetes knowledge	None (theoretical assumptions)
Sowah et al [31]	2020	System design	Ghana	General diabetes knowledge	Technical performance
Anastasiadou et al [32]	2020	System design	Greece	Diet, exercise, medications, blood glucose, treatment, learning resources, and doctor information	User experience
Beaudry et al [33]	2019	Pilot study	United States	Drugs and general knowledge of chronic diseases	User experience
Mitchell et al [34]	2021	Pilot study	United States	Diet and exercise	User experience
Hossain et al [35]	2022	System design	Bangladesh	General diabetes knowledge	Technical performance
Xie et al [6]	2018	System design	China	Diet, medication, and symptoms	Technical performance
Maher et al [36]	2020	Interventional study	Australia	Diet and exercise	User experience and user health
Sagstad et al [37]	2022	Interventional study	Norway	Diet, exercise, blood glucose, and gestational diabetes	User experience
Mash et al [38]	2022	Interventional study	South Africa	Diet, exercise, medications, blood glucose, complications, tobacco, alcohol, and mental health	User experience
Baptista et al [39]	2020	Interventional study	Australia	Diet, exercise, medications, blood glucose, and complications	User experience
Hurmuz et al [40]	2020	Interventional study	Netherlands	Diet, exercise, and social support	User experience and user health
Gong et al [41]	2020	Interventional study	Australia	Diet, exercise, medications, blood glucose, and complications	User experience and user health
Roca et al [42]	2021	Interventional study	Spain	Medicine	User experience and user health
Pimenta et al [43]	2022	System design	Portugal	Diet, exercise, and medication	None (theoretical assumptions)
Balsa et al [44]	2020	Pilot study	Portugal	Diet, exercise, and medication	User experience
Félix et al [45]	2019	System design	Portugal	Diet, exercise, and medication	None (theoretical assumptions)
Saritha et al [46]	2020	System design	India	Diabetes general knowledge	Technical performance
Buinhas et al [47]	2019	Pilot study	Portugal	Diet, exercise, and medication	User experience
Dhinagaran et al [48]	2021	Interventional study	Singapore	Diet, exercise, sleep, and mental health	User experience and user health
Dhinagaran and Car [49]	2022	Pilot study	Singapore	Diet, exercise, sleep, and stress management	User experience
Hussain and Athula [50]	2018	Pilot study	Australia	General knowledge of diabetes	Technical performance and user experience
Rehman et al [51]	2020	Pilot study	South Korea	Disease prediction	Technical performance and user experience

### Characteristics of Included Studies

#### Self-Management Content

Most chatbots provided self-management services (n=17, 68%) around lifestyle interventions (diet and exercise). In addition to providing information on diet and exercise, several studies simultaneously provided information about medications, blood glucose, and complications. In total, 5 (20%) studies provided the management of mental health dimensions such as anxiety, depression, social support, and stress. Two (8%) other studies provided functions in sleep quality, smoking, and alcohol consumption (see Tables S4-6 in Multimedia Appendix 1 for details). The chatbots included in this study provide education and self-management services to different types of populations (including people with type 2 diabetes, adolescents, older adults, etc; see Tables S4-6 in Multimedia Appendix 1 for details). For example, the Tess chatbot is intended for adolescents. Tess is designed to respond to users with scripted statements that are crafted by mental health professionals to mimic empathy and compassion. Similar to how therapists can adapt their style or approach to address each client’s needs, Tess intervenes and responds based on an individual’s reported emotions or concerns [29].

#### Question and Answer Database Source

Researchers have mainly collected and extracted diabetes-related health information as a database source for chatbots through the International Diabetes Federation, clinical guidelines on diabetes published in various countries, authoritative health websites, and medical journal literature (see Tables S4-6 in Multimedia Appendix 1 for details).

#### Interaction and Input-Output Methods

When users interact with a chatbot to ask a question, the chatbot could provide 3 different forms of question and answer: free input (where the user freely enters a question), button input (where the user clicks on a topic or number button to ask a question), and mixed mode (where both free input and button modes are available). Many chatbots support users’ use of unrestricted free speech for queries (n=12, 48%). Ten (40%) studies supported users in making inquiries based on topic buttons provided by the chatbots, and 3 (12%) other studies supported mixed mode. Most chatbots allowed users to enter questions using text (n=19, 76%), and a small number of chatbots allowed users to make inquiries using voice (Tables S4-6 in Multimedia Appendix 1).

#### Research Strategy

In our study, research strategies can be categorized into 3 types: qualitative research (n=9, 36%), quantitative research (n=1, 4%), and mixed research (n=7, 28%). Qualitative and quantitative research, in particular, have their own strengths and weaknesses, and mixed research allows quantitative and qualitative research to complement each other’s strengths (the advantages of quantitative, qualitative, and mixed studies are detailed in Multimedia Appendix 2).

#### The Theoretical Framework

A total of 10 (40%) articles included in our study reported theoretical frameworks (Tables S4-6 in Multimedia Appendix 1). These theoretical frameworks mainly include the Behavior Change Wheel, Self-Determination Theory, Behavior Change Theory (Cross-Theoretical Model and Social Cognitive Theory), and so on. One of the more applied theoretical frameworks in the study was the Behavior Change Wheel, followed by the Behavior Change Theory and the Self-Determination Theory. The researchers integrated the theoretical frameworks in the design and intervention functions of the chatbot (see Multimedia Appendix 3 for specific explanations and applications of the theoretical frameworks in detail).

#### Research Type

In our study, researchers classified all articles into 3 types of studies based on research characteristics: system design studies (n=8, 32%), pilot studies (n=8, 32%), and intervention studies (n=9, 36%). In system design studies, chatbots are at an early stage of the study. Most articles at this stage (n=5, 62.5%) consist of theoretical concepts or design chatbots based on theoretical concepts and test their performance. In pilot studies, researchers go deeper in their exploration based on system design. Articles at this stage choose small-scale, short-term experiments to evaluate the effect of chatbots. The intervention studies were a deeper exploration by the researcher based on the pilot study. This study phase has more participants, a longer intervention period, and a more standardized study design. Specific characteristics of chatbots in each study type are detailed in Multimedia Appendices 1 and 4.

### Meta-Analysis Results

The studies included in the meta-analysis were pre-post trials. Based on the homogeneity characteristics of the included studies, the researchers used HbA_1c_ and weight change as outcome indicators. In the meta-analysis, the researchers used the diabetes chatbot as an intervention to analyze the changes in HbA_1c_ and body weight of the study participants after a period of intervention, compared to the baseline. The results of the meta-analysis are shown below.

#### HbA1c

**Figure 2:**
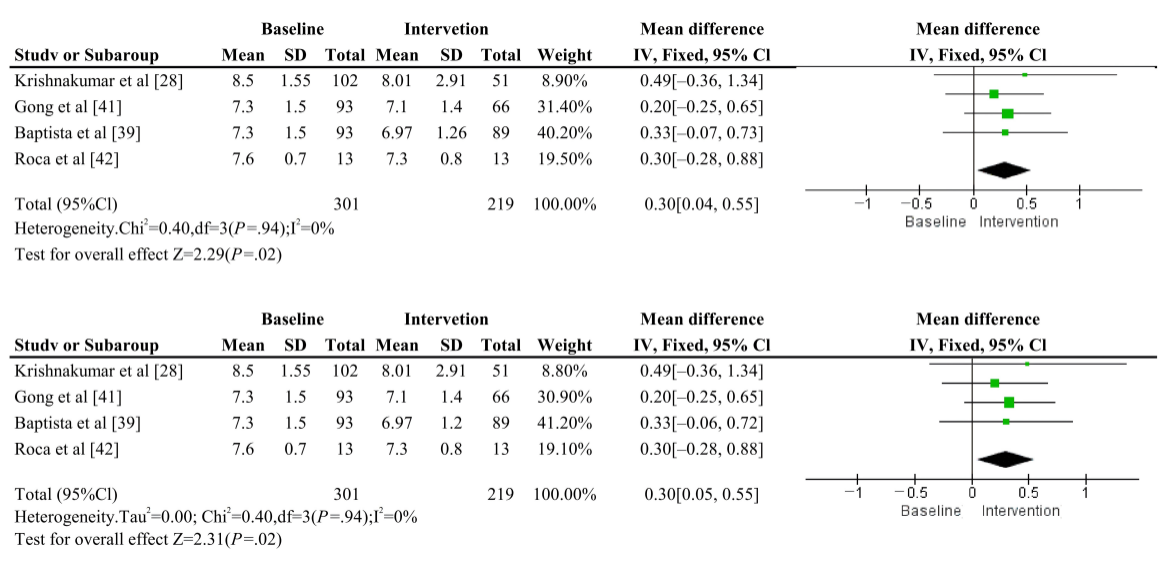
Comparison of fixed-effects and random-effects models in meta-analysis of HbA1c. HbA1c: hemoglobin A1c.

Meta-analysis of HbA_1c_ included 4 articles (Krishnakumar et al [28], Gong et al [41], Baptista et al [39], and Roca et al [42]), totaling 4 groups of pre-post trials and 219 trial participants (Figure 2). The results showed that there was no overall heterogeneity among the 4 groups of included trials (*I*^2^=0%, *P*=.94). The combined total effect size and 95% CI obtained using a fixed-effects model was (mean difference [MD] 0.30, 95% CI 0.04-0.55; *P*=.02), which reached the level of significance. Thus, the chatbot intervention had an effect on lowering HbA_1c_ compared with the baseline. This analysis had consistent results under both fixed-effects and random-effects models. Article publication bias was assessed using funnel plots (Figure S1 in Multimedia Appendix 1).

#### Weight

**Figure 3:**
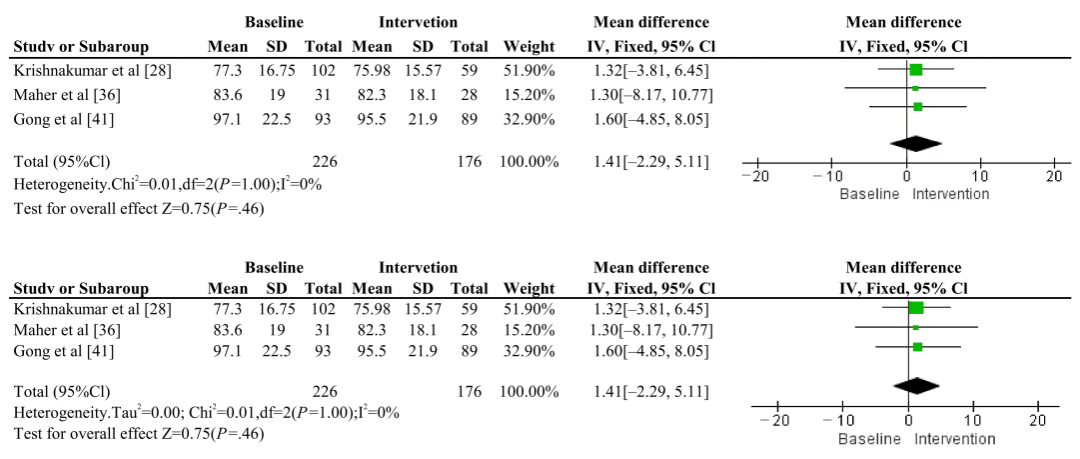
Comparison of fixed-effects and random-effects models in meta-analysis of weight.

Meta-analysis of weight included 3 articles (Krishnakumar et al [28], Maher et al [36], and Gong et al [41]), totaling 3 sets of pre-post trials with 176 trial participants (Figure 3). The results showed that there was no overall heterogeneity among the 3 groups of included trials (*I*^2^=0%, *P*>.99). The combined total effect size and 95% CI obtained using a fixed-effects model was (MD=1.41, 95% CI –2.29 to 5.11; *P*=.46), which did not reach the level of significance. Thus, there was no significant effect of the chatbot intervention in reducing body weight compared to baseline. This analysis had consistent results under both fixed-effects and random-effects models. Article publication bias was assessed using funnel plots (Figure S2 in Multimedia Appendix 1).

## Discussion

### Principal Findings

This study is a systematic review and meta-analysis of the effectiveness of chatbots in diabetes self-management. A total of 25 articles were analyzed in this review. Based on the type of study, the 25 studies can be categorized as systematic design type studies, pilot studies, and intervention studies. Chatbots provide services involving both physical and psychological aspects. The criteria for assessing the effectiveness of chatbots can be categorized into technical performance assessment, user experience assessment, and user health assessment. Overall, the results of the evaluation of the chatbot model were good, and the overall acceptance of chatbots by users was high.

### General Overview of Study

Chatbots could play an important role in future self-management for patients with diabetes. However, the current use of chatbots in diabetes self-management is still in its infancy. The number of publications in each country was low, and there was room for further research and development. Compared to high-income countries, there were fewer publications in low-income countries. However, low-income countries have a large population base, and the number of people with diabetes is increasing. Therefore, researchers should pay attention to conducting relevant research in low-income countries in the future.

The chatbot can provide management on physical dimensions such as diet, exercise, medication, blood glucose, and complications management. Only a few articles provided services on psychological dimensions such as anxiety, depression, stress, and social support. More than half of the diabetes chatbots enable users to enter questions freely. A few chatbots offer button input. More than half of the chatbots offer text-based counseling, and a few chatbots allow for voice-to-text counseling. Voice interaction can facilitate users’ interaction with chatbots. Therefore, this represents an area for future research. Chatbots need to establish an appropriate rapport with the user through personalized and friendly interactions for sustained and engaging interventions.

### Meta-Analysis Results

Conducting a meta-analysis of the interventional studies, the researchers extracted 2 outcome metrics with a high degree of homogeneity—HbA_1c_ and body weight—as criteria for evaluating the effectiveness of the chatbot intervention. Other intervention studies have used measures such as quality of life, BMI, waist circumference, blood pressure, diabetes knowledge, and risk perception. Meta-analysis of the study results showed that the chatbot intervention was effective in lowering blood glucose but had no significant effect on weight reduction. For example, a pilot study conducted over a 4-month period allowed patients to record their meals, weight, physical activity, and blood glucose in a chatbot. Patients received sessions on self-care behaviors and were provided with feedback from the AI-powered chatbot, as well as regular informational interactions with a diabetes educator via voice calls. To increase patient motivation and reduce the difficulty of performing specific tasks, the chatbot facilitates patient action through a variety of forms of brief educational content (e.g., informative lessons, videos, quizzes, and stories). The program uses a gamified approach to build self-management skills and rewards patients with virtual trophies as they complete lessons, tasks, and challenges. As participation in the program increased, patients’ HbA_1c_, fasting blood glucose, and postprandial blood glucose levels gradually decreased [29].

### The Role of Chatbots

The results of this study found that as technologies such as semantic analysis and natural language processing continue to be incorporated into chatbots, researchers have built a knowledge base of diabetes questions and answers with logical rules, so that chatbots can provide users with personalized advice and management services to help patients with diabetes improve their self-management.

Chatbots can help people with diabetes manage their diet, exercise, medication, blood glucose monitoring, prevention of complications, and mental health to control blood glucose levels and improve quality of life [33-35,43-45]. In addition, chatbots also have a significant advantage in out-of-hospital diabetes management. Chatbots can save many human medical resources, reducing the work pressure on primary care staff and the burden on the health care system [52]. Therefore, chatbots with AI technology will play a prominent role in diabetes health education and self-management, which is in line with the trend of digital therapy.

Based on the analysis of the effectiveness assessment indicators (technical performance indicators, user experience, and user metrics) of the chatbot, it was found that the technical assessment of the chatbot model was good [6], the overall acceptance of the chatbot by the users was high [29], and the health status of the users improved after using the chatbot for self-management [36,38,41,42]. Overall, the use of chatbots in health education and self-management of diabetic patients is effective.

### Lack of High-Quality Research Evidence

Identifying the best evidence is an important part of evidence-based medicine. Evidence grading is an essential tool to help decision-makers obtain the best evidence. Although the criteria and methods for grading research evidence are not uniform, there is some grading of evidence that is generally accepted by scientists. These gradings have consistently endorsed meta-analyses and randomized controlled trials (RCTs) as the highest level of evidence [53]. Most of the articles in our study were system design studies and pilot studies that were in the primary and secondary research phases, while fewer intervention studies were in the analysis. Most of the studies in the system design category tested the model performance of chatbots through algorithms and techniques. However, the actual effectiveness of chatbot applications in the self-management of patients with diabetes has yet to be verified at this stage. In the pilot studies, the number of respondents was small (n=10-33), the duration of the intervention was short (from short-term measurements to 24 weeks), and the respondents included older adults and the general public in addition to people with diabetes. Most of the intervention research articles took a pre-post trial design, and only 1 document used an RCT design with the highest level of evidence. There is still a lack of higher level studies, especially RCTs, to evaluate the effectiveness of chatbots in diabetes self-management. RCTs are important for evaluating the effectiveness of chatbots in diabetes self-management. Therefore, more high-quality studies, such as RCTs, should be conducted in the future to evaluate the effectiveness of chatbots in diabetes self-management, providing stronger evidence that will contribute to the development of the field.

### Single Method of Research

Most of the articles included for analysis in our study used quantitative research methods. The data sources mainly relied on data collected from chatbots and user questions and answers, as well as user ratings of the experience, satisfaction, and usability of chatbots collected from questionnaires. In contrast, only a minority of articles used mixed research methods (MMRs) and qualitative research methods (semistructured interviews, focus group discussions, etc). For example, of the pilot studies, most used quantitative research methods, with only 2 MMR studies and 1 qualitative study. The quantitative studies were mainly based on questionnaires and data collected from chatbots, while MMR studies included interviews with users on top of that. MMR has unique advantages [54]. The original purpose of the MMR was to bridge the gap between the quantitative and qualitative paradigms of analysis, combining the strengths of both to allow a broader and deeper analysis of specific issues. Quantitative research is objective and reproducible but does not facilitate the researcher’s observation and communication with the research participants. Qualitative research allows the researcher to observe and communicate with the research participants in a close and more natural setting. Qualitative research makes it easier for the researcher to observe and understand the research participants’ behavior, attitudes, and motivations from the participant’s perspective, and the research design is more flexible. Adding qualitative research to quantitative research allows researchers to better understand what respondents think about chatbots. Respondents may raise issues that the researcher has not noticed and considered, helping the researcher to improve the research design. Through qualitative research, the researcher can fully play into the motivation of the researcher and interviewees by conducting semistructured interviews and focus group discussions with interviewees. The interview questions centered on the features that users want chatbots to have or their feelings when using chatbots. After the interviews, the researcher organizes the collected data and information and analyzes them to gain an in-depth understanding of the users’ experiences, perceptions, and barriers to the use of chatbots so as to provide assistance for subsequent research. Therefore, more MMRs should be used in future research on chatbots and diabetes self-management. By combining the respective strengths and unique perspectives of qualitative and quantitative, a more comprehensive understanding of the impact of chatbots on diabetes self-management can be achieved.

### Insufficiently Innovative Theoretical Framework

In our study, it was found that only a minority of studies had a theoretical framework to support them, while most studies did not have a theoretical framework. For example, only 4 of the pilot studies were guided by a theoretical framework. This phenomenon suggests that researchers have not paid enough attention to the theoretical framework. Theories are developed to explain, predict, and understand phenomena. A scientific theoretical framework actively supports and guides research design and experimentation and can inform and guide the selection of research types. A theoretical framework helps the researcher synthesize prior empirical findings within a theoretical framework, clarifying what the concepts are and how they relate to each other. It provides guidance for research at the methodological level. It helps the researcher to build bridges between theories, facilitates the researcher to recognize their own research, and discovers connections to the established body of knowledge.

The main theoretical frameworks used in the field of diabetes self-management and chatbots include Behavior Change Theory, Self-Determination Theory, Cross-Theoretical Models, and Social Cognitive Theory. These theoretical frameworks are relatively well-developed and widely used. Applying a theoretical framework can provide a theoretical foundation for research on chatbots in diabetes self-management. The use of appropriate theoretical frameworks can enhance understanding of the underlying mechanisms between chatbots and diabetes self-management and facilitate the development of effective interventions. Therefore, future research is expected to use more novel theoretical frameworks in studies related to chatbots and diabetes self-management. Or innovatively applying theoretical frameworks from other fields to enrich research in the field of chatbots and diabetes self-management (see Multimedia Appendix 3 for specific explanations and applications of the theoretical frameworks in detail).

### Equitable Accessibility and Privacy Protection

In addition, although patients with diabetes and those at risk of developing diabetes are getting younger, older adults still account for a high proportion of patients with diabetes.

Chatbots, a new-era product of mobile health and AI, should consider the needs of different age groups in terms of system design and software interface usage. Especially for older adults, their acceptance of the new system, their learning level, whether the software interface is easy to use, whether the font size is comfortable, and whether the answers are easy to understand need special consideration. In order to ensure that digital health tools such as chatbots are accessible and beneficial to everyone, it is crucial to include diverse populations in future research.

At the same time, chatbots collect a large amount of health and personal data from users. If this information is disclosed, it will violate patients’ privacy rights. Lack of privacy protection will affect users’ trust. Chatbots can earn users’ trust through dialog and prompt them to reveal more personal data [55]. Therefore, it is crucial to safeguard patients’ personal privacy. First, regulators must act to ensure user safety and privacy. For example, on March 13, 2024, the AI Bill was passed in the European Union (EU) Parliament and will soon become EU law. The EU’s AI Bill will require high-risk AI systems to be assessed and monitored before approval [56]. Second, operators should standardize the management of data usage rights of back-office personnel and strengthen core data positions and departmental prevention. Third, users should be clearly informed of the privacy policy. The privacy policy must be dynamic, and the privacy policy must provide a table of contents to facilitate users’ understanding of the content of the privacy policy. The collection of users’ personal information shall be based on the principles of clear purpose, explicit consent, and collection of the least necessary data, as well as a detailed description of the type of information to be collected, its purpose, and the manner in which it is to be collected. In addition, operators need to give the correct download uniform resource locator or store. According to their own services, they target the older adults and minors for safety knowledge popularization. When sharing and using personal information, it needs to be deidentified or anonymized so that the subject of the personal information cannot be identified [57].

### Strengths and Limitations

This study is the first systematic review of chatbots and self-management for people with diabetes. Researchers searched for relevant literature from 2 authoritative databases, Web of Science and PubMed. The researchers analyzed and summarized the specific features of chatbots in the field of diabetes, as well as information on the research methodology, research design, and theoretical framework used in the articles. In addition, the articles included from different countries may increase the generalizability of our findings.

However, this article also has some limitations. First, researchers selected more authoritative and comprehensive databases, but some gray literature was not retrieved. Second, in the process of data extraction, the key information extracted was confirmed by experts and extracted and cross-checked by 2 researchers independently, but there was still a possibility of human error. There is some literature that fits the research theme very well, but it was not included in our study because full-text information was not available. Only English-language studies were included in this review, which may lead to a certain degree of publication bias. Finally, the trials in the meta-analysis were all pre-post trials with low evidence validity. Therefore, the effectiveness of chatbot interventions remains to be validated by conducting trials.

### Conclusions

This study analyzes and summarizes information on the specific characteristics of chatbots in the field of self-management of patients with diabetes, such as research methods, research strategies, effectiveness evaluation metrics, etc, and provides suggestions for research related to chatbots and the self-management of patients with diabetes. The number of articles published in each country was low, and chatbots were at an early stage of development in the field of self-management of patients with diabetes. Chatbots provide advice and education focused on the physical aspects of patients, and researchers have focused less on the psychological aspects of patients. A number of positive effects of chatbots in supporting the self-management of people with diabetes. The overall acceptance of chatbots among people with diabetes was high. However, most of the current studies were at the stage of system design and pilot studies. Meta-analysis showed that the chatbot intervention was effective in lowering blood glucose but had no significant effect on weight reduction.

Based on the results derived from this study, we make the following appeal to researchers: (1) focus should be placed on the area of chatbots and diabetes self-management, with research to fully demonstrate the effectiveness of chatbot interventions; (2) future studies should address identified research gaps and use higher level evidence, such as RCTs; (3) in the choice of research strategies, more mixed research should be adopted to fully use the advantages of quantitative and qualitative research; (4) use of appropriate and innovative theoretical frameworks in the study to provide theoretical support for the study; and (5) the digital age requires the protection of users’ private data. It is hoped that researchers will continue to innovate and enrich the research in this field, which will help to continuously develop effective intervention measures of chat robots in diabetes self-management, and ultimately help patients with diabetes stay healthy.

## Appendix

Multimedia Appendix 1 Supplementary tables and figures.

Multimedia Appendix 2 Analysis of the advantages and disadvantages of quantitative, qualitative and mixed studies.

Multimedia Appendix 3 Overview of theoretical frameworks and specific applications to chatbots and diabetes.

Multimedia Appendix 4 Research type.

Multimedia Appendix 5 PRISMA 2020 checklist.

## Abbreviations

AI: artificial intelligence
EU: European Union
HbA1c: hemoglobin A1c
MD: mean difference
MMR: mixed research method
PRISMA: Preferred Reporting Items for Systematic Reviews and Meta-Analyses
RCT: randomized controlled trial

## Data Availability

The data sets generated and analyzed during this study are available from the corresponding author on reasonable request.
