# Supplementary material for: Application of Chatbots to Help Patients Self-Manage Diabetes: Systematic Review and Meta-Analysis
Source: J Med Internet Res. 2024 Dec 3;26:e60380. doi: 10.2196/60380 (PMC11653048; doi:10.2196/60380)
Supplement: Multimedia Appendix 1 [file jmir_v26i1e60380_app1.docx]

**Multimedia Appendix 1**

**Supplementary Tables and Pictures**

**Table S1. Search string**

| **Database** | **Number of articles retrieved** |
| --- | --- |
| **Web of Science** | 2696 |
| TS=((Conversational agent OR conversational agents OR conversational system OR conversational systems OR dialog system OR dialog systems OR dialogue systems OR dialogue system OR assistance technology OR assistance technologies OR relational agent OR relational agents OR chatbot OR chatbots OR digital agent OR digital agents OR digital assistant OR digital assistants OR virtual assistant OR virtual assistants OR Question answering system OR [Question-Answer System](https://kns.cnki.net/kns8/Detail?sfield=fn&QueryID=6&CurRec=10&DbCode=IPFD&dbname=IPFDLAST2022&filename=KZJC202208003043) OR question-response system OR Query Answering System) AND (diabetes Mellitus OR diabetes OR DM OR Type 2 Diabetes Mellitus OR Type 2 Diabetes OR Type 2 Diabetic OR Diabetes type 2 OR T2DM OR Type 1 Diabetes Mellitus OR Type 1 Diabetes OR Type 1 Diabetic OR T1DM)). |  |
| **Pubmed** | 109 |
| ((Conversational agent OR conversational agents OR conversational system OR conversational systems OR dialog system OR dialog systems OR dialogue systems OR dialogue system OR assistance technology OR assistance technologies OR relational agent OR relational agents OR chatbot OR chatbots OR digital agent OR digital agents OR digital assistant OR digital assistants OR virtual assistant OR Question answering system OR Question-Answer System OR question-response system OR Query Answering System )) AND ((diabetes Mellitus OR diabetes OR DM OR Type 2 Diabetes Mellitus OR Type 2 Diabetes OR Type 2 Diabetic OR Diabetes type 2 OR T2DM OR Type 1 Diabetes Mellitus OR Type 1 Diabetes OR Type 1 Diabetic OR T1DM)) |  |

**Table S2.** The inclusion and exclusion criteria for screening articles

| **Inclusion criteria** | **Reason** |
| --- | --- |
| （1）the language type was English | English, one of the most common international languages, was chosen to facilitate the researcher's screening and study. |
| (2) the literature type was research articles | Ensure that articles included in the analysis contain the information needed by the researcher, such as specific research outcomes. |
| (3) the data source was Web of Science or Pubmed | Web of Science is the authoritative and comprehensive database, and Pubmed is the leading database in the field of biomedicine. Higher quality of articles. |
| **Exclusion criteria** | **Reason** |
| (1) duplicate article | Make sure to get a realistic number of articles. |
| (2) literature for which full text is not available, such as conference abstracts, etc. | Insufficient or missing literature data for systematic reviews. |
| (3) unrelated to the topic | It is completely unrelated to the study topic and cannot be analyzed. |

**Table S3. AHRQ Quality Assessment Checklist**

|  | 1)Define the source of information (survey, record review) | 2) List inclusion and exclusion criteria for exposed and unexposed subjects (cases andcontrols) or refer to previous publications | 3) Indicate time period used for identifying patients | 4) Indicate whether or not subjects were consecutive if not population-based | 5) Indicate if evaluators of subjective components of study were masked to other aspects ofthe status of the participants | 6) Describe any assessments undertaken for quality assurance purposes (eg, test/retest ofprimary outcome measurements) | 7) Explain any patient exclusions from analysis | 8) Describe how confounding was assessed and/or controlled | 9) lf applicable, explain how missing data were handled in the analysis | 10) Summarize patient response rates and completeness of data collection | 11) Clarify what follow-up, if any, was expected and the percentage of patients for whichincomplete data or follow-up was obtained | Total scale |
| --- | --- | --- | --- | --- | --- | --- | --- | --- | --- | --- | --- | --- |
| Krishnakumar et al [1] | Yes | No | Yes | Yes | Unclear | Yes | Yes | No | Yes | Yes | Yes | 8 |
| Stephens et al [2] | Yes | No | No | Unclear | Unclear | Yes | No | No | No | Yes | No | 3 |
| Beaudry et al [3] | Yes | No | Yes | No | Unclear | Yes | No | No | No | Yes | Yes | 5 |
| Mitchell et al [4] | Yes | No | No | No | No | Yes | No | No | Yes | Yes | Yes | 5 |
| Maher et al [5] | Yes | Yes | Yes | Unclear | No | Yes | Yes | No | Yes | Yes | Yes | 8 |
| Sagstad et al [6] | Yes | No | Yes | No | No | Yes | No | No | No | Yes | No | 4 |
| Mash et al [7] | Yes | No | Yes | Unclear | No | Yes | Yes | No | Yes | Yes | No | 7 |
| Baptista et al [8] | Yes | Yes | Yes | Unclear | No | Yes | Yes | Yes | No | Yes | Yes | 9 |
| Hurmuz et al [9] | Yes | Yes | Yes | Unclear | Unclear | Yes | No | No | No | Yes | Yes | 6 |
| Gong et al [10] | Yes | Yes | Yes | Unclear | Unclear | Yes | Yes | Yes | Yes | Yes | Yes | 9 |
| Roca et al [11] | Yes | Yes | Yes | Unclear | No | Yes | Yes | No | Yes | Yes | No | 7 |
| Balsa et al [12] | Yes | No | No | Unclear | Unclear | Yes | No | No | No | Yes | Unclear | 3 |
| Buinhas et al [13] | Yes | No | No | Unclear | No | Yes | No | No | No | Yes | No | 3 |
| Dhinagaran et al [14] | Yes | No | Yes | Yes | Unclear | Yes | Yes | No | Yes | Yes | Yes | 8 |
| Dhinagaran and Car [15] | Yes | Yes | Yes | Unclear | No | No | Yes | No | No | Yes | Yes | 6 |
| Hussain and Athula [16] | Yes | Yes | No | Unclear | Unclear | Yes | No | No | No | Yes | No | 4 |
| Rehman et al [17] | Yes | No | No | No | Unclear | Yes | No | No | No | Yes | No | 3 |

*Article guality was assessed as follows: low quality = 0-3; moderate quality = 4-7; high quality = 8-11.

**Table S4. Articles characteristics of system design**

| **First Author** | **Research Object** | **Chatbot Name** | **Theoretical framework** | **AI technology** | **Questions and answers source** | **Question form^b^** | **Input mode** | **Output mode** |
| --- | --- | --- | --- | --- | --- | --- | --- | --- |
| Sharma et al [18] | Diabetic Patients | SMDIoT(Smart Data Mining and the Internet of Things) | None | Data mining,  semantic analysis, Machine learning | None | Free Input | Text | Text, Speech |
| Sowah et al [19] | Diabetic Patients | Diabetes Management System | None | Natural Language Processing, Machine learning | Health websites, medical experts | Free Input | Text | Text |
| Anastasiadou et al [20] | Diabetic patients | Education Virtual Assistant | None | Natural Language Processing、Machine learning | Official institutions | Free Input | Text | Text |
| Hossain et al [21] | Diabetic patients | Dm-Health application | None | Natural Language Processing, Machine learning | Health websites, official institutions | Free Input | Text, Speech | Text，Speech |
| Xie et al [22] | Diabetic patients | Dia-AID | None | Semantic Analysis | Health websites, Medical guides | Free Input | Text | Text |
| Pimenta et al [23] | Diabetic patients (type II, elderly) | Victoria(Anthropomorphic Conversational Agent) | Self-Determination Theory (SDT) | Natural Language Understanding，Machine learning，synthetic voice | None | Button input | Text | Text, Speech |
| Félix et al [24] | Diabetic patients (type II, elderly) | victoria | Behavior Change Wheel (BCW) | Natural Language Understanding，Machine learning，synthetic voice | None | Button input | Text | Text, Speech |
| Saritha et al [25] | Diabetic patients | Medibot | None | Natural Language Processing, Natural Language Understanding, Machine learning | None | Free Input | Text | Text |

a: Technical Performance Metrics: Objective assessment of the technical performance of the chatbot system; User experience metrics: Investigate users' feelings and evaluations in the process of experiencing the chatbot through quantitative or qualitative methods; User health indicators: To investigate the changes in health indicators such as blood glucose, weight, and diet scores of users before and after using the chatbot.

b: Free input: the user can enter questions in the chatbot in a free language; Button input: the user needs to click on the topic buttons (number buttons) provided by the chatbot to ask and answer questions; Mixed input: conversations between the user and the chatbot can be entered by a mix of both free-language and topic button approaches.

**Table S5. Article characteristics of the pilot studies**

| **First author** | **Chatbot Name** | **Research Strategy** | **Research Object** | **Theoretical Framework** | **Number of researchers** | **Intervention time** | **Mean age**  **(age range)** | **Measurement method** | **AI technology** | **Question form** | **Input mode** | **Output mode** |
| --- | --- | --- | --- | --- | --- | --- | --- | --- | --- | --- | --- | --- |
| Stephens et al [2] | Tess | Quantitative Research | Teenagers at risk of diabetes | Cognitive Behavioral Therapy | 23 | 10-12 weeks | 15.20 years old | Questionnaires, systematic data collection | Machine learning | Free Input | Text | Text |
| Beaudry et al [3] | SMS Chatbot | Quantitative research | Teenagers with chronic diseases | None | 13 | 24 weeks | Not mentioned (14-17 years old) | Systematic data collection | None | Button input | Text | Text |
| Mitchell et al [4] | t2.coach scripted chatbot | Mixed research | Diabetic patients (type II) | Brief Action Plan | 23 | 2 weeks | 54.92 | Questionnaires, interviews | None | Free Input | Text | Text |
| Balsa et al [12] | victoria | Mixed research | Diabetic patients (type II, elderly) | Behavior Change Wheel | 20^a^ | 3.7 weeks | 70.91 years (patients)/50.43 years (specialists) | Questionnaires, interviews | Natural Language Understanding, Machine learning，synthetic voice | Button Input | Text | Text, Speech |
| Buinhas et al [13] | victoria | Quantitative research | Diabetic patients (type II, elderly) | Behavior Change Wheel | 10^b^ | Short-term measurement | 30.8 years old | Questionnaires | Natural Language Understanding，Machine learning，synthetic voice | Button input | Text | Text, Speech |
| Dhinagaran and Car [15] | Precilla | Qualitative research | Adult public | None | 20 | 4 weeks | 33.0 years old | Questionnaires, interviews | None | Button input | Text | Text, Picture |
| Hussain and Athula [16] | VDMS(Virtual Diabetes Management System) | Quantitative research | Adult public | None | 10 | Short-term measurement | Not mentioned (20-50 years old) | Questionnaires, systematic data collection | Natural Language Processing | Free Input | Text | Text |
| Rehman et al [17] | MIRA (Virtual Medical Assistant) | Quantitative Research | Adult public | None | 33 | Short-term measurement | Not mentioned (18-43 years old) | Questionnaires, systematic data collection | Speech recognition, Natural Language Processing, Natural Language Understanding, Machine learning,Natural Language Understanding | Free Input | Text, Speech | Text, Speech |

a: 11 older adults with type 2 diabetes and 9 specialists.

b: Nurses with primary care expertise.

**Table S6. Article characteristics of the intervention studies**

| **First author** | **Chatbot Name** | **Research Strategy** | **Research type** | **Control group** | **Research Object** | **Theoretical Framework** | **Number of researchers** | **Intervention time** | **Mean age** | **Measurement method** | **AI technology** | **Question form** | **Input mode** | **Output mode** |
| --- | --- | --- | --- | --- | --- | --- | --- | --- | --- | --- | --- | --- | --- | --- |
| Krishnakumar et al [1] | Wellthy CARE | Quantitative Research | Non-Randomized Controlled Trials | Before and after self-control | Diabetic patients (Type II, adults) | Digital persuasion model | 102 | 16 weeks | 50.8 years old | Systematic data collection, physical measurement data | None | Free input | Text, Speech | Text, Speech |
| Maher et al [5] | Paola | Mixed research | Non-Randomized Controlled Trials | Before and after self-control | Middle-aged and elderly (45-75 years old) | None | 31 | 12  weeks | 56.2 years old | Questionnaires, systematic data collection, physical measurements | Natural Language Processing | Free input | Text | Text |
| Sagstad et al [6] | Dina information chat robot | Quantitative Research | Large data studies | Uncontrolled | Diabetic patients (Pregnancy) | None | none | 20  weeks | None | Systematic data collection | None | Mixed input | Text | Text |
| Mash et al [7] | 4Diabetes Whats App | Mixed research | Non-randomized controlled trials | Uncontrolled | Diabetic patients (type II) | None | 575 | 24  weeks | None | Systematic data collection, interview | None | Mixed input | Text, Speech, Picture | Text, Speech, Picture |
| Baptista et al [8] | Laura | Mixed research | Non-randomized controlled trials | Uncontrolled | Diabetic patients (Type II, Adults) | None | 93 | 48  weeks | 55 years old | Questionnaires, interviews | Speech recognition | Button input | Text, Speech | Text, Speech |
| Hurmuz et al [9] | COUCH | Mixed research | Study design | pretest-posttest design | Diabetic patients (Type II, Adult) | Self-determination theory | 50 | 20-36weeks | None | Questionnaires, interviews | None | Button input | Text | Text |
| Gong et al [10] | Laura | Quantitative research | RCT | Routine nursing group | Diabetic patients (Type II, Adults) | Behavior change theory | 187 | 48  weeks | 57 years old | Questionnaires, systematic data collection, physical measurements | Speech recognition | Button input | Text, Speech | Text, Speech |
| Roca et al [11] | Virtual assistant | Mixed research | Non-randomized controlled trials | Uncontrolled | Diabetic patients (Type II) | None | 18^a^ | 36  weeks | 63.8  years old | Questionnaires, interviews, physical measurement data | None | Mixed input | Text | Text, Picture |
| Dhinagaran et al [14] | Precilla | Quantitative research | Non-randomized controlled trial | Uncontrolled | Adult public | Behavior change wheel | 60 | 4  weeks | 33.7  years old | Questionnaires, systematic data collection | None | Button input | Text | Text, picture |

a：5 healthcare professionals and 13 patients with type 2 diabetes and depression


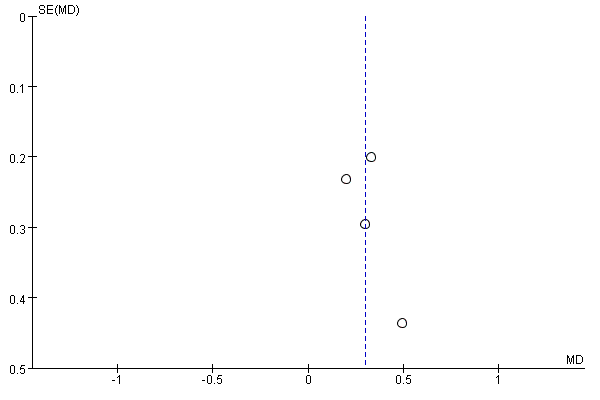


**Figure S1. HbA1c Meta-Analysis Funnel Plot**


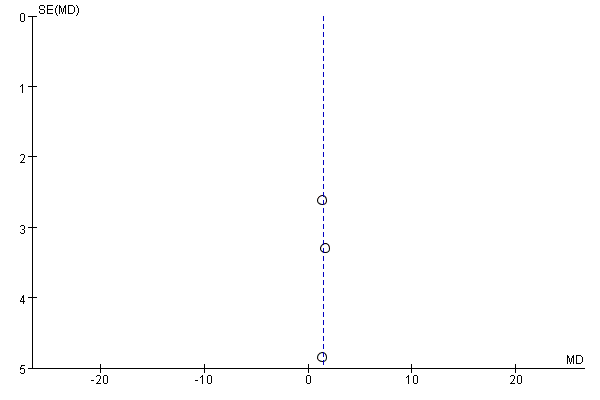


**Figure S2. Weight Meta-Analysis Funnel Plot**

**References**

1.Krishnakumar A, Verma R, Chawla R, Sosale A, Saboo B, Joshi S, et al. Evaluating glycemic control in patients of South Asian origin with type 2 diabetes using a digital therapeutic platform: analysis of real-world data. J Med Internet Res. 2021;23(3):e17908. [doi: 10.2196/17908] [Medline: 33764306]

2.Stephens TN, Joerin A, Rauws M, Werk LN. Feasibility of pediatric obesity and prediabetes treatment support through tess, the AI behavioral coaching chatbot. Transl Behav Med. 2019;9(3):440-447. [doi: 10.1093/tbm/ibz043] [Medline: 31094445]

3. Beaudry J, Consigli A, Clark C, Robinson KJ. Getting ready for adult healthcare: designing a chatbot to coach adolescents with special health needs through the transitions of care. J Pediatr Nurs. 2019;49:85-91. [doi: 10.1016/j.pedn.2019.09.004] [Medline: 31644960]

4. Mitchell EG, Maimone R, Cassells A, Tobin JN, Davidson P, Smaldone AM, et al. Automated vs. human health coaching: exploring participant and practitioner experiences. Proc ACM Hum Comput Interact. 2021;5(CSCW1):99. [doi: 10.1145/3449173] [Medline: 36304916]

5. Maher CA, Davis CR, Curtis RG, Short CE, Murphy KJ. A physical activity and diet program delivered by artificially intelligent virtual health coach: proof-of-concept study. JMIR Mhealth Uhealth. 2020;8(7):e17558. [doi: 10.2196/17558] [Medline: 32673246]

6. Sagstad MH, Morken N, Lund A, Dingsør LJ, Nilsen ABV, Sorbye LM. Quantitative user data from a chatbot developed for women with gestational diabetes mellitus: observational study. JMIR Form Res. 2022;6(4):e28091. [doi: 10.2196/28091] [Medline: 35436213]

7. Mash R, Schouw D, Fischer AE. Evaluating the implementation of the GREAT4Diabetes WhatsApp chatbot to educate people with type 2 diabetes during the COVID-19 pandemic: convergent mixed methods study. JMIR Diabetes. 2022;7(2):e37882. [doi: 10.2196/37882] [Medline: 35537057]

8. Baptista S, Wadley G, Bird D, Oldenburg B, Speight J, My Diabetes Coach Research Group. Acceptability of an embodied conversational agent for type 2 diabetes self-management education and support via a smartphone app: mixed methods study. JMIR Mhealth Uhealth. 2020;8(7):e17038. [doi: 10.2196/17038] [Medline: 32706734]

9. Hurmuz MZM, Jansen-Kosterink SM, Op den Akker H, Hermens HJ. User experience and potential health effects of a conversational agent-based electronic health intervention: protocol for an observational cohort study. JMIR Res Protoc. 2020;9(4):e16641.[doi: 10.2196/16641] [Medline: 32242517]

10. Gong E, Baptista S, Russell A, Scuffham P, Riddell M, Speight J, et al. My diabetes coach, a mobile app-based interactive conversational agent to support type 2 diabetes self-management: randomized effectiveness-implementation trial. J Med Internet Res. 2020;22(11):e20322. [doi: 10.2196/20322] [Medline: 33151154]

11. Roca S, Lozano ML, García J, Alesanco Á. Validation of a virtual assistant for improving medication adherence in patients with comorbid type 2 diabetes mellitus and depressive disorder. Int J Environ Res Public Health. 2021;18(22):12056.[doi: 10.3390/ijerph182212056] [Medline: 34831811]

12. Balsa J, Félix I, Cláudio AP, Carmo MB, Silva ICE, Guerreiro A, et al. Usability of an intelligent virtual assistant for promoting behavior change and self-care in older people with type 2 diabetes. J Med Syst. 2020;44(7):130. [doi: 10.1007/s10916-020-01583-w] [Medline: 32533367]

13. Buinhas S, Cláudio AP, Carmo MB, Balsa J, Cavaco A, Mendes A, et al. Virtual assistant to improve self-care of older people with type 2 diabetes: first prototype. In: Gerontechnology. Cham, Switzerland. Springer; 2019:236-248.

14. Dhinagaran DA, Sathish T, Soong A, Theng Y, Best J, Tudor Car L. Conversational agent for healthy lifestyle behavior change: web-based feasibility study. JMIR Form Res. 2021;5(12):e27956. [doi: 10.2196/27956] [Medline: 34870611]

15. Dhinagaran DA, Car LT. Public perceptions of a healthy lifestyle change conversational agent in Singapore: a qualitative study. Digit Health. 2022;8:20552076221131190. [doi: 10.1177/20552076221131190] [Medline: 36267545]

16. Hussain S, Athula G. Extending a conventional chatbot knowledge base to external knowledge source and introducing user based sessions for diabetes education. 2018. Presented at: 32nd International Conference on Advanced Information Networking and Applications Workshops (WAINA); May 16-18, 2018:698-703; Krakow, Poland. URL: https://ieeexplore.ieee.org/abstract/document/8418155

17. Rehman UU, Chang DJ, Jung Y, Akhtar U, Razzaq MA, Lee S. Medical instructed real-time assistant for patient with glaucoma and diabetic conditions. Appl Sci. 2020;10(7):2216. [doi: 10.3390/app10072216]

18. Sharma M, Singh G, Singh R. An advanced conceptual diagnostic healthcare framework for diabetes and cardiovascular disorders. EAI Endorsed Trans Scalable Inf Syst. 2018;5(18):154828.

19. Sowah RA, Bampoe-Addo AA, Armoo SK, Saalia FK, Gatsi F, Sarkodie-Mensah B. Design and development of diabetes management system using machine learning. Int J Telemed Appl. 2020;2020:8870141. [doi: 10.1155/2020/8870141] [Medline: 32724304]

20. Anastasiadou M, Alexiadis A, Polychronidou E, Votis K, Tzovaras D. A prototype educational virtual assistant for diabetes management. 2020. Presented at: IEEE 20th International Conference on Bioinformatics and Bioengineering (BIBE); October 26-28, 2020:999-1004; Cincinnati, OH. URL: https://ieeexplore.ieee.org/abstract/document/9288129

21. Hossain E, Alshehri M, Almakdi S, Halawani H, Rahman MM, Rahman W, et al. Dm-Health app: diabetes diagnosis using machine learning with smartphone. Comput Mater Continua. 2022;72(1):1713-1746.

22. Xie W, Ding R, Yan J, Qu Y. A mobile-based question-answering and early warning system for assisting diabetes management. Wirel Commun Mob Comput. 2018;2018(s1):1-14. [doi: 10.1155/2018/9163160]

23. Pimenta N, Félix IB, Monteiro D, Marques MM, Guerreiro MP. Promoting physical activity in older adults with type 2 diabetes an anthropomorphic conversational agent: development of an evidence and theory-based multi-behavior intervention. Front Psychol. 2022;13:883354. [doi: 10.3389/fpsyg.2022.883354] [Medline: 35903740]

24. Félix IB, Guerreiro MP, Cavaco A, Cláudio AP, Mendes A, Balsa J, et al. Development of a complex intervention to improve adherence to antidiabetic medication in older people using an anthropomorphic virtual assistant software. Front Pharmacol. 2019;10:680. [doi: 10.3389/fphar.2019.00680] [Medline: 31281256]

25. Saritha AK. Medibot A predictive generic diabetic chatbot using bagging ensemble hybrid learning. Int J Eng Adv Technol. 2020;9(4):2249-8958.
