# Supplementary material for: Application of Chatbots to Help Patients Self-Manage Diabetes: Systematic Review and Meta-Analysis
Source: J Med Internet Res. 2024 Dec 3;26:e60380. doi: 10.2196/60380 (PMC11653048; doi:10.2196/60380)
Supplement: Multimedia Appendix 2 [file jmir_v26i1e60380_app2.docx]

**Multimedia Appendix 2**

**Analysis of the advantages and disadvantages of quantitative, qualitative and mixed studies**

**1. Quantitative research**

Quantitative research adopts a positivist paradigm, using quantitative means (numbers and statistics) to understand communication phenomena. Its research aims to identify correlations and causal relationships, and thus its conclusions are precise and generalized. It provides information about a phenomenon or thing at the macro level and has credibility. Common quantitative research methods include surveys, experiments and content analysis. The characteristics of quantitative research include (1) the pursuit of "larger sample sizes, better statistical significance." It emphasizes the objectivity and reproducibility of the study. (2) The research process measures objective facts, and the researcher is divorced from the study, thus the research process is not influenced by personal factors[1].

**2 Qualitative research**

Qualitative research is the process of gaining keen insights by uncovering problems, understanding the phenomena of events, analyzing human behavior and perspectives, and answering questions. Qualitative research is a study of a small, carefully selected group of sample individuals. It does not require statistical significance, but with the researcher's experience, sensitivity, and relevant techniques, it can be effective in gaining insights into the behavior and motivations of the subjects, the impact they may have, and so on. Characteristics of qualitative research include:

(1) The researcher observes and communicates with the research subjects in a close and more natural environment, which is conducive to observing and understanding their behaviors, attitudes, motivations, etc.; (2) Research design is more flexible; (3) Its findings tend to leave a lot of room for interpretation; (4) Individuals play a large role in the research process, fully mobilizing the participants.

**3 Mixed research**

Mixed Methods Research (MMR) emphasizes pragmatism and is the third research paradigm that organically combines the two major research paradigms of quantitative research and qualitative research[2]. This method can help researchers to solve some problems that cannot be completely, reasonably and comprehensively explained by qualitative or quantitative alone, and maximize the realization of the research purpose. MMR is advantageous:

(1) Improving the accuracy of research results. MMR provides the researcher with an opportunity to validate the results of the study with different methods.

(2) Provide a more complete description. Quantitative research perspectives are drawn from closed data, while qualitative research perspectives are drawn from open personal data. MMR tends to look at the problem both quantitatively and qualitatively, obtaining two different perspectives, thus combining the two approaches to provide a more complete explanatory perspective.

(3) Complementing each other's strengths and enhancing complementary advantages. Quantitative and qualitative research have their relative strengths and weaknesses, and the value of using mixed methods is to utilize the strengths of one method to compensate for the inherent weaknesses or biases of a particular method.

**References**

1. Johnson RB, Onwuegbuzie AJ. Mixed methods research: a research paradigm whose time has come. Educ Res. 2004;33(7):14-26. [doi: 10.3102/0013189X033007014]
2. Pierre P .Critical appraisal tools for assessing the methodological quality of qualitative, quantitative and mixed methods studies included in systematic mixed studies reviews.J Eval Clin Pract. 2013;19(4):722.[doi: 10.1111/jep.12017]
