# Supplementary material for: Application of Chatbots to Help Patients Self-Manage Diabetes: Systematic Review and Meta-Analysis
Source: J Med Internet Res. 2024 Dec 3;26:e60380. doi: 10.2196/60380 (PMC11653048; doi:10.2196/60380)
Supplement: Multimedia Appendix 3 [file jmir_v26i1e60380_app3.docx]

**Multimedia Appendix 3**

**Overview of theoretical frameworks and specific applications to chatbots and diabetes**

The Behavior Change Wheel (BCW)[1] provides a structured approach to behavioural interventions. The core of the Behavior Change Wheel is a model of human behaviour, the COM-B. It views human behaviour (B) as the result of the interaction of physical and psychological capacities (C), opportunities provided by the physical and social environment (O), and reflection and motivation (M). The BCW categorises the content and components of behavioural interventions into nine intervention functions (IFs): constraints, environmental restructuring, modelling, enabling, training, coercion, incentives, persuasion and education[2].

Two studies[2,3]used the Behavior Change Wheel as a theoretical framework to explore the usability of intelligent virtual assistants in promoting behaviour change, self-care and medication adherence in elderly patients with type 2 diabetes. The researchers derived behavior change techniques (BCTs) for improving medication adherence based on BCW in three stages. Stage 1: Understanding the Behavior. Researchers address this stage by reviewing the evidence on medication adherence and its determinants, including barriers and facilitators to medication use. The researchers then mapped these determinants to COM-B and identified key factors that have a greater impact on behavior change. Stage 2: Identifying Intervention Functions. The same IF can be linked to different COM-B domains. The IFs most likely to influence behavior change are 1) affordability, 2) utility, 3) effectiveness and cost-effectiveness, 4) acceptability, 5) side effects/safety, and 6) equity. Stage 3: Stage 3: Identifying Behavior Change Techniques. BCTs are known as the effective components of interventions. BCTs include a total of 93 techniques, such as education, training, and persuasion. The researchers analyzed to determine the most common BCTs under each IF and effective interventions for medication adherence.

Self-determination theory (SDT) is a broad meta-theory of motivation. Central to the theory is the distinction between autonomous motivation (intrinsic motivation) and controlled motivation (external motivation)[4]. Autonomous motivation is the motivation to pursue a goal or take action when a person has sufficient will and through a self-approved choice[5]. In contrast, controlled motivation refers to a passive action taken as a result of some form of pressure, whether external pressure from others or internal pressure from the individual. It has been shown that more autonomous motivation, as opposed to controlled motivation, is not only associated with a variety of positive outcomes, but is critical to those positive outcomes. A study [5] linked behaviour change techniques to self-determination theory to provide theoretical support for the design of studies using anthropomorphic conversation agents to promote physical activity in older adults with type 2 diabetes. The researcher is committed to increasing autonomous motivation in patients and improving physical activity in people with diabetes by meeting the three basic psychological needs of human autonomy, relatedness, and competence, ultimately achieving optimal intervention outcomes, psychological health, and well-being.

An interactive anthropomorphic conversation agent——Laura[6,7]guides users through dialogue scripts and algorithms designed by applying behaviour change theory and technology. These include transtheoretical models[8], social cognitive theory[9], gamification[10] and concepts from chronic disease self-management education[11]. The transtheoretical model of behaviour change[8] is considered a useful framework for helping people with diabetes to change their behaviour[12]. This model describes five stages of change that individuals go through when changing their behaviour: pre-contemplation, contemplation, preparation, action and maintenance[13]. Therefore, the transtheoretical model can provide a staged intervention programme. One study found that stage-matched intervention programmes were more effective than non-stage-matched intervention programmes because they addressed the needs of all individuals at different stages of change. Therefore, matching intervention strategies to patients' stages of change can increase the likelihood of regular participation in a diabetes intervention programme and prevent patients from dropping out[14].

Social Cognitive Theory(SCT) is one of the most commonly used models and theories to improve self-management behaviour in patients with chronic conditions[15]. SCT theory suggests that self-management education in diabetes and pre-diabetes patients is in a dynamic state of change as it interacts with personal and environmental factors. Among the behavioral factors are motivation, level of effort, progress, efficiency and adherence to self-management education. The more motivated a patient is, the more likely they are to put in more effort and the more efficient they are at self-management education. A patient's self-efficacy is his or her internal confidence in overcoming various barriers to adherence to self-management education. Positive outcome expectations are manifested in the conviction that self-management education facilitates glycemic control. Patients attribute success to their own abilities, efforts, choices, and use of strategies that contribute to self-management education. Patients with strong self-assessment and regulation skills promote self-management education by analyzing available resources (space, equipment, etc.) and their own strengths (ability, knowledge, and experience, etc.), selecting appropriate types of self-management education, setting quantifiable, easy-to-difficult goals, developing a specific plan, and continually adjusting the plan. Environmental factors include role modeling, guidance and social feedback. Role modeling by close friends, guidance from professionals, support and appreciation from family and friends, and equipment, and knowledge are conducive to patients' adherence to self-management. Behavioral factors, environmental factors, patient attributions, self-assessment, and regulation work together to influence patients' self-efficacy and outcome expectations, affecting thinking patterns, motivation, and affective responses to self-management education for patients with diabetes, and consequently, the choice to begin, persist in or stop self-management.

**References**

1. Michie S, Atkins L, West R. The behavior change wheel: a guide to designing interventions. Great Britain: Silverback Publishing. 2015.
2. Balsa J, Félix I, Cláudio AP, et al. Usability of an intelligent virtual assistant for promoting behavior change and self-care in older people with type 2 diabetes. J Med Syst. 2020;44(7):1-12.
3. Félix IB, Guerreiro MP, Cavaco A, et al. Development of a complex intervention to improve adherence to antidiabetic medication in older people using an anthropomorphic virtual assistant software. Front pharmacol. 2019;10:680.
4. Ryan RM, Deci EL. Self-determination theory: Basic psychological needs in motivation, development, and wellness. Guilford Publications. 2017.
5. Pimenta N, Félix IB, Monteiro D, et al. Promoting physical activity in older adults with type 2 diabetes via an anthropomorphic conversational agent: development of an evidence and theory-based multi-behavior intervention. Front Psychol. 2022;4108.
6. Baptista S, Wadley G, Bird D, et al. Acceptability of an embodied conversational agent for type 2 diabetes self-management education and support via a smartphone app: mixed methods study. JMIR mHealth uHealth. 2020;8(7):e17038.
7. Gong E, Baptista S, Russell A, et al. My Diabetes Coach, a mobile app–based interactive conversational agent to support type 2 diabetes self-management: randomized effectiveness-implementation trial. J Med Internet Res. 2020;22(11): e20322.
8. Prochaska JO, Redding CA, Evers KE. The Transtheoretical model and stages of change. Health Educ Behav. 1997;22(22):97-121.
9. Islam KF, Awal A, Mazumder H, et al. Social cognitive theory-based health promotion in primary care practice: A scoping review. Heliyon. 2023;9(4):e14889.
10. Cugelman B. Gamification: what it is and why it matters to digital health behavior change developers. JMIR Serious Games. 2013;1(1):e3.
11. Grady PA, Gough LL. Self-management: a comprehensive approach to management of chronic conditions. Am J Public Health. 2014;104(8):e25-e31.
12. Honda H, Igaki M, Tanaka SI, et al. Impact of Self-Reported Sitting Time and Transtheoretical Model Based on Exercise Behavior Change on Glycemic and Weight Control in Japanese Adults with Type 1 Diabetes: A Cross-Sectional Study. Healthcare. 2020;8(2):105.
13. Prochaska JO, DiClemente CC, Norcross JC. In search of how people change:applications to addictive behaviors. Am Psychol. 1992;47:1102-14
14. Kim C J, Hwang A R, Yoo J S. The impact of a stage-matched intervention to promote exercise behavior in participants with type 2 diabetes. Int J Nurs Stud. 2004;41(8):833-841.
15. Zhao F, Suhonen R, Koskinen S, et al. Theory-based self-management educational interventions on patients with type 2 diabetes: a systematic review and meta-analysis of randomized controlled trials. J Adv Nurs. 2017;73(4):812-833.
