# Supplementary material for: Application of Chatbots to Help Patients Self-Manage Diabetes: Systematic Review and Meta-Analysis
Source: J Med Internet Res. 2024 Dec 3;26:e60380. doi: 10.2196/60380 (PMC11653048; doi:10.2196/60380)
Supplement: Multimedia Appendix 4 [file jmir_v26i1e60380_app4.docx]

**Multimedia Appendix 4**

**Research type**

In our study, researchers classified all articles into three types of studies based on research characteristics: system design studies (n=8), pilot studies (n=8), and intervention studies (n=9).

(1) In system design studies, chatbots are at an early stage of the study. Most articles at this stage consist of theoretical concepts or design chatbots based on theoretical concepts and test their performance.

(2) In pilot studies, researchers go deeper in their exploration based on system design. Most articles at this stage choose small-scale, short-term experiments to evaluate the effect of chatbots.

(3) The intervention study is a deeper exploration by the researcher based on the pilot study. This study phase has more participants, a longer intervention period, and a more standardised study design.

Specific characteristics of chatbots in each study type are detailed in Supplementary Appendix A and Appendix D

***System design study***

The articles in the system design category (Supplementary Appendix A) mostly described the specific design of the chatbot and the technologies used, and evaluated the technical performance of the chatbot. This type of article provided a more detailed description of the AI technologies and algorithms used in chatbots. AI technologies mainly included natural language processing, machine learning, speech recognition, etc. Algorithms include neural networks, support vector machines, random forests, evolutionary algorithms, plain Bayes, decision trees, and K-nearest neighbour algorithm. Model evaluation indexes can reflect part of the model's performance. A reasonable selection of evaluation metrics can accurately evaluate the model performance, and, at the same time, discover the problems of the model itself, so as to guide the design and optimization direction of the model. For system design types of studies, their evaluation metrics were mostly technical performance evaluation metrics such as precision[1], recall[1], usability[2], acceptability[2], accuracy[3], and so on. For example, accuracy refers to the number of correct information bars extracted/number of information bars extracted during the evaluation of the chatbot model. Recall refers to the number of correct message bars extracted/number of message bars in the sample. Most of the authors reported good evaluation results. These results indicate that the chatbots designed by researchers have value for further development.

***Pilot study***

The pilot study of articles (Supplementary Appendix A) mostly recruited participants (patients, experts, or the public) to experience and evaluate the chatbots. However, the number of participants in this type of article was small, and the intervention time was short. The majority of pilot studies used user experience indicators (such as usability, engagement, usefulness, satisfaction, etc.), and only a few studies also evaluated the technical performance of chatbots (such as accuracy in answering users' questions). No studies had addressed user health evaluation metrics (such as Blood glucose and weight change). Most authors found the results of the chatbot's user experience evaluation to be favorable, but in one study, users also suggested improvements to the chatbots[2].

***Intervention study***

The interventional study articles (Supplementary Appendix A) were more in-depth, evidence-efficient studies based on systems design and pilot studies. The subjects in this type of study were mostly patients with diabetes and were recruited in larger numbers and for longer interventions than in the pilot studies. Among the interventional studies, more articles used non-randomised controlled trials (n=6), and only one article used a randomized controlled trial[4]. The metrics used to evaluate the effects in the intervention research articles mostly used user experience and user health evaluation metrics. User experience evaluation metrics included technology acceptance[5], system ease of use[5], willingness to pay[5], applicability[6,7], satisfaction[6,7], and so on. User health evaluation indicators included HbA1c change, physical activity time, diet score, height, weight, BMI, waist circumference, blood pressure, quality of life, self-management education ability, sleep, stress, etc. (see Supplementary Appendix A).

**References**

1. Xie W, Ding R, Yan J, Qu Y. A mobile-based question-answering and early warning system for assisting diabetes management. Wirel Commun Mob Comput. 2018;2018(s1):1-14. [doi: 10.1155/2018/9163160]
2. Sowah RA, Bampoe-Addo AA, Armoo SK, Saalia FK, Gatsi F, Sarkodie-Mensah B. Design and development of diabetes management system using machine learning. Int J Telemed Appl. 2020;2020:8870141.[doi: 10.1155/2020/8870141] [Medline: 32724304]
3. Hossain E, Alshehri M, Almakdi S, Halawani H, Rahman MM, Rahman W, et al. Dm-Health app: diabetes diagnosis using machine learning with smartphone. Comput Mater Continua. 2022;72(1):1713-1746.
4. Gong E, Baptista S, Russell A, Scuffham P, Riddell M, Speight J, et al. My diabetes coach, a mobile app-based interactive conversational agent to support type 2 diabetes self-management: randomized effectiveness-implementation trial. J Med Internet Res. 2020;22(11):e20322.[doi: 10.2196/20322] [Medline: 33151154]
5. Hurmuz MZM, Jansen-Kosterink SM, Op den Akker H, Hermens HJ. User experience and potential health effects of a conversational agent-based electronic health intervention: protocol for an observational cohort study. JMIR Res Protoc. 2020;9(4):e16641.[doi: 10.2196/16641] [Medline: 32242517]
6. Mash R, Schouw D, Fischer AE. Evaluating the implementation of the GREAT4Diabetes WhatsApp chatbot to educate people with type 2 diabetes during the COVID-19 pandemic: convergent mixed methods study. JMIR Diabetes. 2022;7(2):e37882.[doi: 10.2196/37882] [Medline: 35537057]
7. Dhinagaran DA, Sathish T, Soong A, Theng Y, Best J, Tudor Car L. Conversational agent for healthy lifestyle behavior change: web-based feasibility study. JMIR Form Res. 2021;5(12):e27956.[doi: 10.2196/27956] [Medline: 34870611]
